# Cost-effectiveness of molecular diagnostic assays for the therapy of severe sepsis and septic shock in the emergency department

**DOI:** 10.1371/journal.pone.0217508

**Published:** 2019-05-24

**Authors:** Ioannis M. Zacharioudakis, Fainareti N. Zervou, Fadi Shehadeh, Eleftherios Mylonakis

**Affiliations:** 1 Infectious Diseases Division, Warren Alpert Medical School of Brown University, Rhode Island Hospital, Providence, Rhode Island, United States of America; 2 Division of Infectious Diseases and Immunology, Department of Medicine, NYU School of Medicine, New York, New York, United States of America; Azienda Ospedaliero Universitaria Careggi, ITALY

## Abstract

**Objectives:**

Sepsis presents a major burden to the emergency department (ED). Because empiric inappropriate antimicrobial therapy (IAAT) is associated with increased mortality, rapid molecular assays may decrease IAAT and improve outcomes. We evaluated the cost-effectiveness of molecular testing as an adjunct to blood cultures in patients with severe sepsis or septic shock evaluated in the ED.

**Methods:**

We developed a decision analysis model with primary outcome the incremental cost-effectiveness ratio expressed in terms of deaths averted. Costs were dependent on the assay price and the patients’ length of stay (LOS). Three base-case scenarios regarding the difference in LOS between patients receiving appropriate (AAT) and IAAT were described. Sensitivity analyses regarding the assay cost and sensitivity, and its ability to guide changes from IAAT to AAT were performed.

**Results:**

Under baseline assumptions, molecular testing was cost-saving when the LOS differed by 4 days between patients receiving IAAT and AAT (ICER -$7,302/death averted). Our results remained robust in sensitivity analyses for assay sensitivity≥52%, panel efficiency≥39%, and assay cost≤$270. In the extreme case that the LOS of patients receiving AAT and IAAT was the same, the ICER remained≤$20,000/death averted for every studied sensitivity (i.e. 0.5–0.95), panel efficiency≥34%, and assay cost≤$313. For 2 days difference in LOS, the bundle approach was dominant when the assay cost was≤$135 and the panel efficiency was≥77%.

**Conclusions:**

The incorporation of molecular tests in the management of sepsis in the ED has the potential to improve outcomes and be cost-effective for a wide range of clinical scenarios.

## Introduction

Sepsis presents a major burden to U.S. emergency departments (ED), with up to 850,000 estimated visits annually between 2009–2011 [1]. Treatment of septic patients places a significant financial burden on the U.S. healthcare system with estimated $20.3 billion spent in 2011 [2], and the annual rate of increase of the average cost of hospital stay for sepsis is three-times the rate for hospital costs overall [3].

The management of sepsis relies on early initiation of appropriate antimicrobial therapy (AAT) [4], with a goal of administering AAT within 1 hour of recognition of severe sepsis or septic shock [5, 6]. While sepsis-related mortality rate can exceed 40% [7], empiric inappropriate antimicrobial therapy (IAAT) for severe infections has been shown to increase 30-day mortality by 71% and in-hospital mortality by 67% [8]. To improve outcomes, policymakers are increasingly using regulatory mechanisms intended to provide incentives to clinicians and hospitals to improve the quality of sepsis care, such as the “Rory’s Regulations” in New York State [9]. Initial antimicrobial therapy is currently empiric, based on the clinical syndrome, patient risk factors and local antimicrobial resistance profile. The rapid identification of pathogens for targeted antimicrobial therapy has been limited by the use of blood cultures, that need 48–72 hours for microbe isolation [10, 11]. Rapid molecular diagnostic techniques for the identification of bloodstream pathogens directly from whole blood samples have been developed recently, with the potential of pathogen identification in 2–7 hours [12].

The importance of early initiation of AAT, and the observed reluctance of providers to adjust antimicrobial therapy initiated in the ED when they assume patient care in the inpatient setting [13], highlights the importance of the ED in sepsis management. In this study, we constructed a decision analysis to study the cost-effectiveness of implementing a molecular assay as an adjunct to blood cultures upon presentation of a patient with severe sepsis or septic shock in the ED.

## Methods

**Fig 1:**
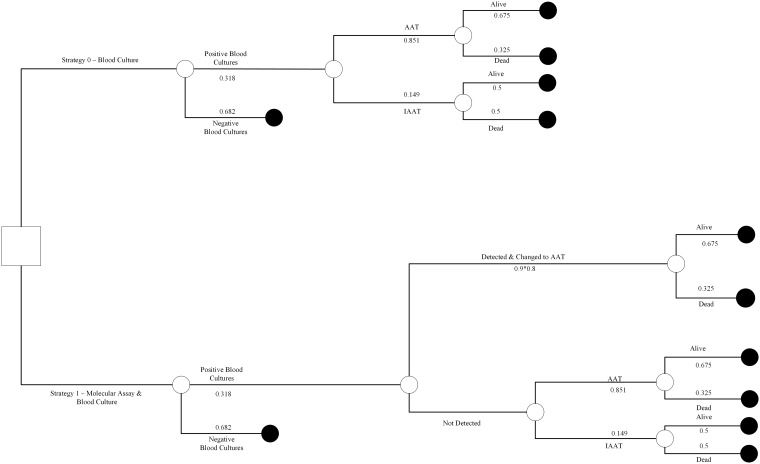
Decision tree with base case input values. The decision tree is a graphical display of a logical sequence of events in the two study arms. The *square* represents the decision node from which the two competing strategies (molecular method plus blood cultures *vs*. blood cultures alone) originate. The *circles* are chance nodes that lead to a particular outcome (e.g. survival or death) beyond the control of our decision. The probabilities assigned to the decision tree are for baseline analysis and are listed in Table 1.

We designed a decision analysis model to examine the cost-effectiveness of a bundle approach that involves collection of both a rapid diagnostic molecular test and blood cultures at the time of presentation of a patient with severe sepsis or septic shock in the ED (Fig 1). Data for the model were extrapolated from the literature as described below.

### A. Model structure

There were 2 interventions entailed in the described model. The first included the collection of 2 sets of blood cultures from patients who presented to the ED with severe sepsis or septic shock, as per standard of care. In the second arm, patients had also molecular testing performed at the same time with blood cultures. Patients could receive AAT or IAAT. Therapy was considered appropriate when the causative pathogens were sensitive *in vitro*. In cases of polymicrobial infections, all pathogens that were felt to be contributing to severe sepsis or septic shock had to be covered by antibiotics to which the organisms were sensitive *in vitro* [14]. The decision tree with the base case input values is presented in Fig 1.

### B. Assigning probabilities and costs

The assigned probabilities and costs, as extrapolated from current literature, are displayed in Table 1. Based on the study of Gaieski *et al*. [14], a cohort study of patients who presented with severe sepsis or septic shock in the ED of an academic tertiary center between 2005 through 2006, 25.7% of infections were caused by Gram-positive organisms, 36.6% by Gram-negative organisms, and 2.3% by fungi. In this study, severe sepsis and septic shock were defined based on the 2003 criteria [15]. Based on the results of the same study, 85.1% of patients who were tested with blood cultures received AAT, while 14.9% received IAAT. Moreover, based on the study’s mortality data, patients who were treated with AAT in the ED had a 67.5% chance of survival to hospital discharge, compared to 50% for those who initially received IAAT [14]. The difference in hospital length of stay (LOS) between patients receiving AAT and IAAT differed between studies. Wilke *et al*. reported 4 days difference in LOS (23.9 *vs*. 28.3 days) in patients with hospital-acquired or ventilator-associated pneumonia [16]. Marschall *et al*. found no difference in LOS in patients with Gram-negative bacteremia [17], whereas Shorr *et al*. reported a difference of 2 days in patients with Gram-negative sepsis [18]. Given the variation in the estimations [8, 17–21], three base case scenarios regarding the difference in the hospital LOS between patients receiving AAT and IAAT were described, i.e. 0, 2 or 4 days.

**Table 1:** Model inputs and baseline estimates.

Model Variable	Value	Source
**Percentage of positive blood cultures**	0.318	[14]
**Percentage of isolated Gram-positive bacteria**	0.257	[14]
**Percentage of isolated Gram-negative bacteria**	0.366	[14]
**Percentage of isolated fungi**	0.023	[14]
**Overall percentage of AAT**	0.851	[14]
**Survival rate of patients receiving AAT**	0.675	[14]
**Survival rate of patients receiving IAAT**	0.500	[14]
**Sensitivity of molecular assay**	0.90 [0.5–0.95]	[22]
**Panel efficiency of molecular assay**	0.67 [0.3–0.9]	Calculated from [14]
**Difference in Length of Stay AAT and IAAT**	[0–4] d	[17, 18, 20]
**Cost of molecular assay**	$130 [$100 -$1,000]	[25]
**Hospitalization Cost per day**	$2367.01	[23]

AAT = Appropriate Antimicrobial Therapy, d = days, IAAT = Inappropriate Antimicrobial Therapy

On the base-case scenario the molecular method had a 90% sensitivity of detecting the pathogen that would eventually grow in the blood culture. This was based on the sensitivity of the currently FDA approved molecular assays [22]. Blood culture, the gold standard reference test for the purposes of this analysis, was assumed to have a 100% sensitivity and specificity. Based on the study by Gaieski *et al*., in 68.2% of patients, blood cultures finalized negative and antimicrobial therapy was considered neither appropriate or inappropriate [14]. This percentage includes patients with other positive cultures rather than blood (e.g. patients with positive urine cultures), since in those cases the blood culture, and as such the molecular testing results, would not be used to guide therapy.

On the base case analysis, the molecular test results led to a change of therapy in 2/3 of cases on IAAT, i.e. based on the molecular test results 66.7% of patients who were initially on IAAT would change to AAT (defined as panel efficiency). This was assumed to be achieved by the detection of microbes that were not covered by the initial empiric treatment (i.e. fungal infections), or had resistance to the empiric therapy based on the hospital antibiogram or the detected resistance genes (e.g. *mec*, *vanA* and *vanB*, or extended-spectrum beta-lactamase (ESBL)). For example, the identification of microbes with unique antimicrobial resistance profiles, such as the *Stenotrophomonas maltophila*, would guide the change from IAAT to AAT. The results of the molecular testing were provided within 2–7 hours of testing allowing timely adjustment of treatment. We assumed that the patient who was eventually treated appropriately adopted the survival rate and the hospital LOS of patients treated with AAT given the rapid turn-around time of the molecular assay.

The cost of hospitalization was estimated using the data by the Kaiser Family Foundation and was adjusted to 2017 US dollars based on the cumulative inflation rate ($2,271 per day of hospitalization that is $2,367.01 adjusted to 2017 US dollars) [23, 24]. The cost of the molecular test at the base case scenario was assumed to be $155 [25]. The costs of the testing machine and labor were considered to be part of the cost of the molecular assay. For the patients that did not grow any microbes in their blood cultures, the cost of the molecular testing was added in the intervention arm with no effect on patients’ survival or hospital LOS.

### C. Outcomes and data analysis

The primary outcome of the base case analysis was the incremental cost-effectiveness ratio (ICER), in terms of deaths averted, among the 2 competing strategies. ICER was measured as the excess cost of a competing strategy divided by the incremental difference in survival. The incremental costs were estimated taking into account the differences in attributed costs to molecular testing and the hospital LOS based on the appropriateness of the antimicrobial therapy.

After the base-case evaluations, we accounted for uncertainty using one-way sensitivity deterministic methods to examine the robustness of our results. Point estimates regarding the sensitivity of the molecular method, the cost of the molecular method and the panel efficiency were adjusted to the predefined extremes as presented in Table 1. Also, to account for the uncertainty that all patients who were changed from IAAT to AAT based on the molecular test results were able to adopt the survival rate and the hospital LOS of patients treated with AAT from their presentation to the ED, we calculated the ICER per antimicrobial therapy that was changed from inappropriate to appropriate. The Mathworks Matlab R2017a was used for the design and analysis of the cost-effectiveness model. Please refer to the S1 Appendix to see the equations used for this analysis.

## Results

We evaluated three base case scenarios in which the hospital LOS differed by 0, 2 and 4 days between patients on AAT and IAAT, as detailed in the *Methods*. In the base case scenarios, the bundle approach that included the simultaneous collection of blood cultures and molecular assay upon presentation to the ED had an estimated cost of $6,929, $7,019 and $7,109 per patient for a difference in LOS of 0, 2 and 4 days, respectively. The relevant costs when only blood cultures were collected were $6,774, $6.999 and $7,223 per patient.

Under the baseline assumptions, the use of the molecular diagnostic method was less costly and more effective in the case that the LOS differed by 4 days between patients receiving AAT and IAAT (ICER -$7,302/death averted and -$599/change from IAAT to AAT). In the case that the LOS was the same (ICER $9,902/death averted and $812/change from IAAT to AAT), or differed only by 2 days (ICER $1,300/death averted and $107/change from IAAT to AAT), the use of the molecular test as an adjunct to the blood cultures was more effective but costlier, with an estimated ICER of ≤$20,000/death averted in both cases.

**Fig 2:**
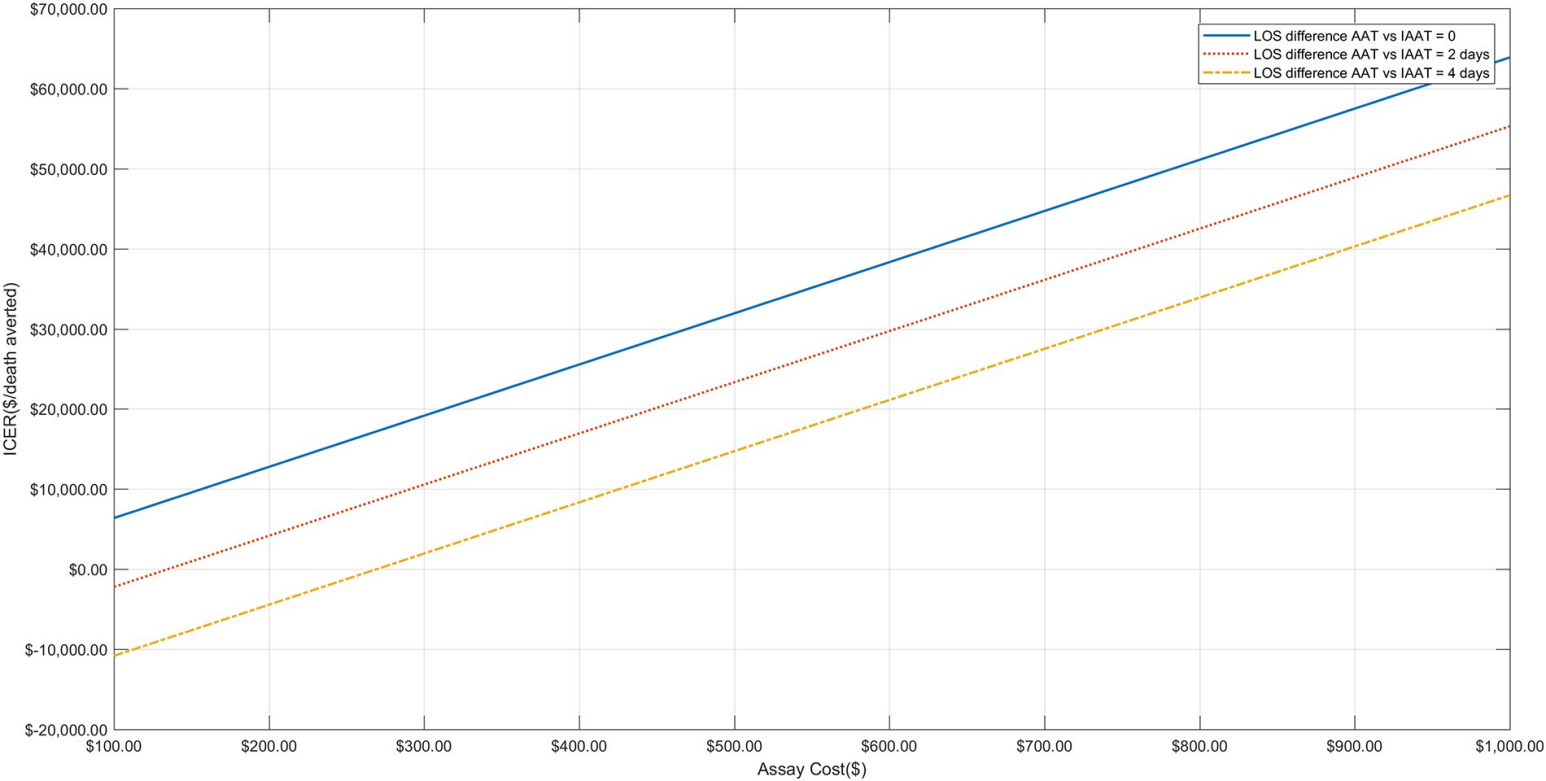
One-way sensitivity analysis for assay cost. One-way sensitivity analysis when assay cost ranges from $100–1,000 for all 4 base case scenarios (i.e. when the difference in hospital length of stay between patients receiving appropriate and inappropriate antimicrobial therapy is 0, 2 and 4 days).

**Fig 3:**
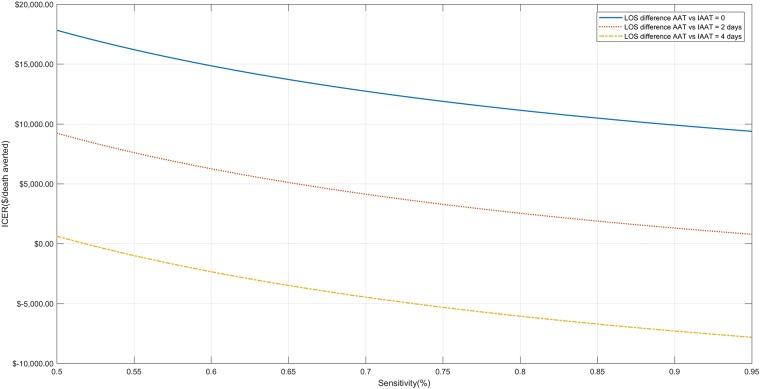
One-way sensitivity analysis for assay sensitivity. One-way sensitivity analysis when the sensitivity of the assay ranges from 50%-95% for all 4 base case scenarios (i.e. when the difference in hospital length of stay between patients receiving appropriate and inappropriate antimicrobial therapy is 0, 2 and 4 days).

**Fig 4:**
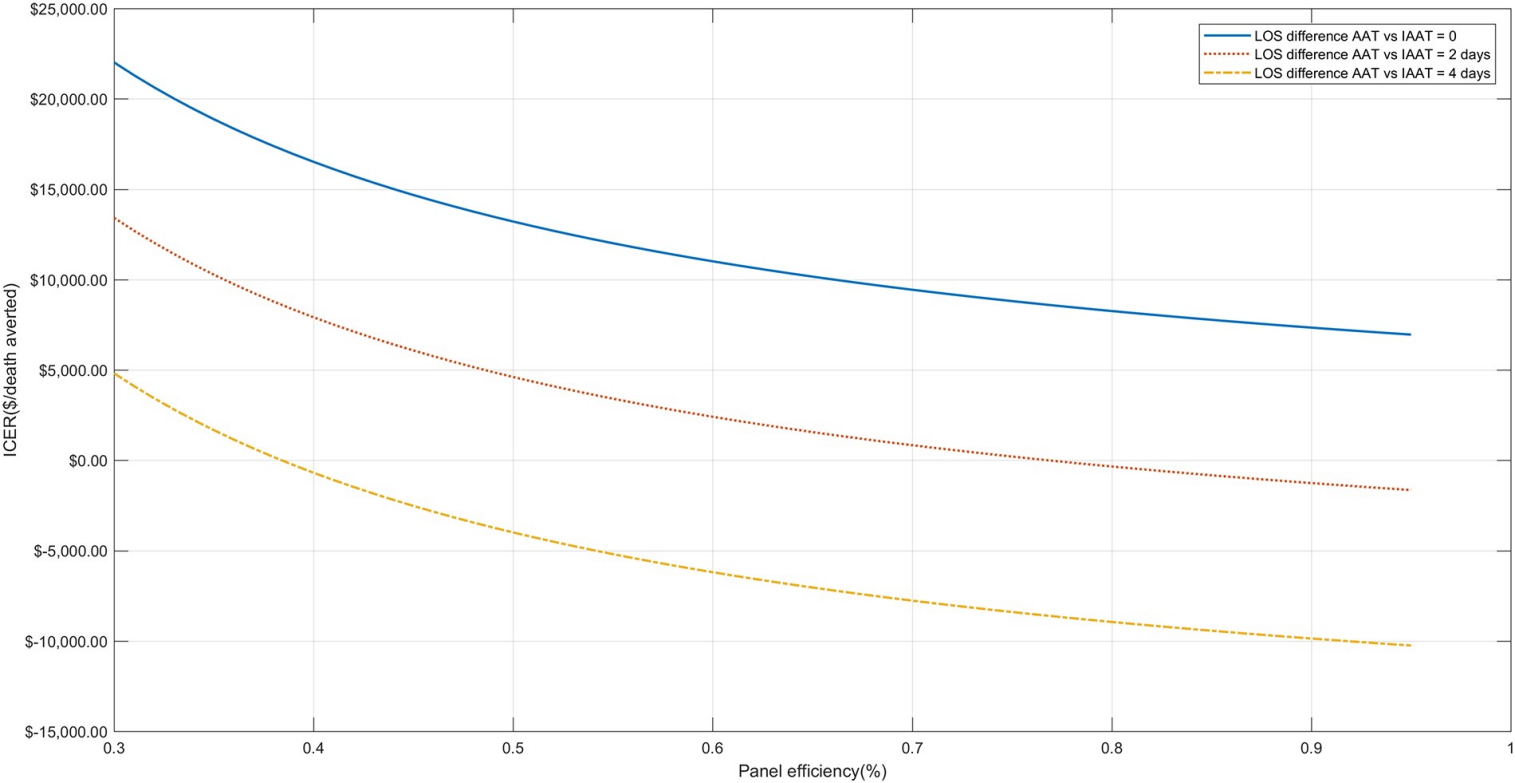
One-way sensitivity analysis for assay efficiency. One-way sensitivity analysis when the efficiency of the assay ranges from 30%-95% for all 4 base case scenarios (i.e. when the difference in hospital length of stay between patients receiving appropriate and inappropriate antimicrobial therapy is 0, 2 and 4 days.

In one-way sensitivity analyses, the dominance of the use of the bundle approach when the LOS differed by 4 days remained when the sensitivity of the molecular assay was ≥52%, the panel efficiency was ≥39%, and the cost was ≤$270, as shown in Figs 2–4. Even in the extreme case that the patients receiving AAT and IAAT had exactly the same length of stay, the ICER remained ≤$20,000/death averted for every studied sensitivity in the pre-specified range (i.e. 0.5–0.95), panel efficiency ≥34%, and assay cost ≤$313. When the difference at the hospital LOS was 2 days, the bundle approach was dominant when assay cost was ≤$135 and the panel efficiency ≥77%. Finally, for assay cost between $135-$447, all examined values of panel efficiency (0.3–0.95) and all examined assay sensitivities (0.5–0.95) the ICER was ≤$20,000/death averted.

## Discussion

In this study, we examined the cost-effectiveness of the use of a rapid molecular diagnostic test for patients who present with severe sepsis or septic shock in the ED. Under baseline assumptions, we found that this bundle is cost-saving in cases that the length of hospital stay differs by 4 days between patients receiving AAT and IAAT. Our results remained robust in sensitivity analyses for assay sensitivity ≥52%, panel efficiency ≥39%, and cost ≤$270. Even in the extreme scenario where the LOS was the same between patients receiving AAT and IAAT, the use of the molecular test as an adjunct to blood cultures remained cost-effective for a willingness to pay ≤$20,000 per death averted for every examined assay sensitivity (0.5–0.95), panel efficiency ≥34%, or cost ≤$313.

Molecular assays with the ability to detect bloodstream pathogens directly in whole blood samples, are highly appealing in the effort of timely, directed treatment in sepsis. In the last decades, there has been an evolution in molecular biology, in regard to the techniques of nucleic acid extraction and amplification, with a growing number of molecular assays becoming available for use in daily clinical practice [12, 22, 26–28]. Septifast is a multiple broad-range real-time polymerase chain reaction (PCR) assay that detects 6 Gram-positive, 8 Gram-negative bacteria, and 5 fungi along with the *mecA* resistance gene within 3.5–5 hours, and is currently in clinical use in Europe [29]. The T2Candida panel is another FDA-approved automated magnetic resonance-based molecular assay that directly identifies *Candida* spp. from whole blood samples with a reported clinical sensitivity of 89% [30]. Multiple clinical trials are now active to either validate the clinical performance of new molecular diagnostic assays (e.g. the T2Bacteria panel, ClinicalTrials.gov Identifier NCT02535468), or to examine the impact of the available molecular assays in patient outcomes, healthcare costs and antibiotic use (ClinicalTrials.gov Identifier NCT03255759). The implementation of such assays as an adjunct to blood cultures at the time of presentation of septic patients to the ED would be expected to maximize the cost-benefit of this bundle approach.

As the IAAT has been shown to increase 30-day mortality by 71% [8], the implementation of a rapid molecular diagnostic test early in the disease course has the potential to significantly improve outcomes by allowing early targeted antimicrobial therapy. EDs provide an appealing setting for the implementation of this bundle to maximize the benefits for each individual patient by minimizing the hours of IAAT. Also, the ED is the place where the initial care is provided to all patients who are admitted from the community, allowing this way to maximize the number of patients that this approach will be implemented to. The rapid de-escalation of the antimicrobial therapy, and the long-term decrease in antimicrobial resistance rates, are also expected to be achieved by the rapid detection of the microbiologic pathogen, and are anticipated to contribute to the decrease in hospital costs and the increase in effectiveness, but these remain to be proven in future studies.

Based on the characteristics of the currently FDA-approved molecular diagnostic techniques, we assumed that the pathogen identification will be done within 2–7 hours [12], allowing the physician to make timely changes in antimicrobial therapy. Studies have suggested that even an hour of delay of AAT can affect mortality [31]. Therefore, even with the assumption that the workflow is optimal, and that the results can be available as early as within 2 hours from presentation to the ED, the mortality might not be exactly the same with that of the patients who were started on AAT from the beginning. To address this uncertainty, we calculated the cost-effectiveness ratio per antimicrobial therapy changed from inappropriate to appropriate. The results of this analysis indicated that even with this measurement of the effectiveness, the implementation of the bundle approach remained appealing and was cost-saving when the difference in the hospital length of stay between patients on AAT and IAAT was 4 days. The above benefit might be maximized by using these molecular diagnostic tests as point-of-care diagnostics in the ED.

The effectiveness of the bundle approach that includes molecular testing is expected to maximize in hospital settings with high prevalence of drug resistant pathogens, and high-risk patients for invasive fungal infections, because of the higher rates of empiric IAAT in this patient population. In order to account for the expected difference in the effectiveness, and subsequently the cost-effectiveness of the bundle approach in this scenario, we performed a sensitivity analysis for an expected change from IAAT to AAT between 30%-95%. Our results indicated that for an at least 2 days difference in LOS between patients on AAT and IAAT, the collection of the molecular diagnostic assay in the ED was cost-effective for a willingness to pay ≤$20,000/death averted for every value of panel efficiency in the aforementioned range. Even when the LOS was the same between patients on AAT and IAAT, the bundle approach remained cost-effective for a willingness to pay of ≤$20,000/death averted for panel efficiency of at least 34%.

For the purpose of this study, we used the 2003 definitions for sepsis and septic shock [15], given that this was the definition used from all the studies that we extrapolated the data for our analysis [14]. However, a 2016 task force proposed a new definition of sepsis [7]. Further studies are needed to determine how the updated definition would affect the results of our analysis. Also, it should be noted that our estimated survival outcomes were based on observational studies [14, 17, 18, 20], and not randomized controlled trials. As such, future randomized trials are needed to confirm the observations of this cost-effectiveness study.

In conclusion, the significant morbidity and mortality of severe sepsis and septic shock have stretched the need for improvement in the quality of sepsis care and there is a need for diagnostic tests with rapid turnaround time that can guide treatment within the first hours of patient presentation to the ED. In an effort to contain the overall cost of medical care, the adaptation of those methods in daily practice is going to be dependent on their cost-efficiency profile. Our analysis provides an estimation of the economic burden of a diagnostic algorithm that incorporates molecular tests in the ED, the first setting where patient present. In this study, we demonstrated that, on baseline scenarios and provided thresholds for willingness to pay of less than $20,000 per death averted, such diagnostic algorithms remain cost-effective even when there is no difference in the LOS between patients on AAT and IAAT, and for a wide range of assay sensitivity, cost and panel efficiency. Clinical trials are needed to prove the robustness of these results.

## Supporting information

S1 Appendix Equations used for the cost-effectiveness study. (PDF)
