# Supplementary material for: Cost-effectiveness of molecular diagnostic assays for the therapy of severe sepsis and septic shock in the emergency department
Source: PLoS One. 2019 May 24;14(5):e0217508. doi: 10.1371/journal.pone.0217508 (PMC6534337; doi:10.1371/journal.pone.0217508)
Supplement: S1 Appendix — (PDF) [file pone.0217508.s001.pdf]

Supplementary Appendix

Equations Used for the Cost-Effectiveness Study

In the baseline arm, only blood cultures were collected and the survival rate was computed based on the percentage of patients receiving AAT and the survival rate of patients on AAT and IAAT.

$$s_o = Perc_{AAT} \cdot Surv_{AAT} + (1 - Perc_{AAT}) \cdot Surv_{IAAT}$$

The cost of this intervention was calculated based on the probability of a patient to receive AAT in the ED, the estimated hospital LOS of patients on AAT and IAAT and the hospitalization cost per day.

$$cost_{o'} = perc_{AAT} \cdot LOS_{AAT} \cdot cost_{hosp/day} + (1 - perc_{AAT}) \cdot LOS_{IAAT} \cdot cost_{hosp/day}$$

The final cost was computed by taking into account that a percentage of the initial blood cultures will finalize negative, as described above.

$$cost_o = perc_{pos} \cdot cost_{o'}$$

In the interventional arm, molecular testing was performed at the time of presentation to the ED as an adjunct to blood cultures in patients diagnosed with severe sepsis or septic shock. The effectiveness of the strategy was defined based on the sensitivity of the assay and the panel efficiency.

$$eff = sensitivity \cdot panelEfficiency$$

The survival rate was presumed to be equal to the survival rate of patients receiving AAT for the patients that were changed from IAAT to AAT based on the molecular assay results, and equal to the survival rate of the baseline arm for the rest.

$$s_1 = eff \cdot Surv_{AAT} + (1 - eff) \cdot s_o$$

Cost of this method was computed based on the hospital LOS for AAT, the effectiveness of the strategy, the hospitalization cost per day and the cost of the assay.

$$cost_{1'} = perc_{AAT} \cdot LOS_{AAT} \cdot cost_{hosp/day} \; +$$

$$+(1 - perc_{AAT}) \cdot (LOS_{IAAT} \cdot (1 - eff) + LOS_{AAT} \cdot eff) \cdot cost_{hosp/day} + cost_{assay}$$

The final cost was computed by taking into account that a percentage of the initial blood cultures finalized negative.

$$cost_1 = perc_{pos} \cdot cost_{1'} + (1 - perc_{pos}) \cdot cost_{assay}$$

Finally, the ICER per death averted and per patient who was changed from IAAT to AAT (ICER') were computed.

$$ICER = \frac{cost_1 - cost_o}{s_1 - s_o}$$

$$ICER' = \frac{cost_1 - cost_o}{perc_{pos} \cdot eff}$$
